# Preventing overfilling of blood bags to reduce clot formation and improve donor safety

**DOI:** 10.1111/vox.70293

**Published:** 2026-05-21

**Authors:** Kabiru M. Sulaiman, Halima Ismail, Yahaya Yazid Ibrahim, Kathleen Selleng, Aisha K. Gwarzo, Dalhat Gwarzo, Andreas Greinacher

**Affiliations:** ^1^ Department of Haematology and Blood Transfusion Aminu Kano Teaching Hospital Kano Nigeria; ^2^ National Blood Service Agency Abuja Nigeria; ^3^ Institut für Transfusionsmedizin, Universitätsmedizin Greifswald Greifswald Germany; ^4^ Medical School of Bayero University Kano Nigeria

**Keywords:** blood bag overfilling, blood donation, clot formation, resource‐limited settings, transfusion safety

## Abstract

**Background and Objectives:**

Overfilling of blood bags is common in low‐resource settings, causing clot formation, donor and recipient risks and blood wastage. This study assessed overfilling prevalence and evaluated a low‐cost intervention to mitigate it.

**Materials and Methods:**

We conducted a prospective study across six blood banks in Kano, Nigeria, weighing 131 blood bags and examining clot formation in 688 consecutive units. A simple, manual measuring and mixing (MeMi) device was developed and implemented to prevent overfilling.

**Results:**

Before intervention, 129/131 blood bags (98.5%) exceeded the 612 g upper limit (mean: 711 g; range: 560–888 g). Systematic assessment revealed macroclots in 72/688 bags (10.5%; 95% confidence interval [CI]: 8.18–12.76). After MeMi use, 658/723 bags (91%) met the target weight (500–612 g), while 2 were overweight (629 g, 639 g) and 63 underweight (265–499 g). Clot incidence dropped to 1.9% (95% CI: 0%–5.1%).

**Conclusion:**

Overfilling of blood bags poses serious safety concerns in low‐resource settings. The MeMi device provides a practical, low‐cost mechanical solution without needing electricity. Locally producible for ~€15, it significantly reduces overfilling and clotting. This improves safety for donors and recipients. Its simplicity and affordability make it highly scalable across regions like sub‐Saharan Africa.

**Highlights**

- Clots in blood bags due to overfilling of blood bags are very frequent in sub‐Saharan Africa due to the lack of automated weighing systems.
- Blood donation volumes >500 mL increase the risk for adverse vasovagal reactions in blood donors and clot formation, causing risks for transfusion recipients.
- Here we present validation data of a highly effective, low‐cost (~€15), manual measuring and mixing (MeMi) device to prevent overfilling of blood bags.

## INTRODUCTION

Blood transfusion is a life‐saving intervention in modern health care, yet access to safe and sufficient blood remains a critical challenge in low‐ and middle‐income countries (LMICs). In sub‐Saharan Africa, the shortage of safe blood is exacerbated by poor infrastructure, limited donor recruitment and inadequate blood safety practices [1, 2]. The World Health Organization (WHO) estimates that 60% of the global population lacks access to safe blood, with the highest burden in LMICs [3].

One of the most common and preventable causes of blood wastage and donor complications in these settings is overfilling of blood bags. While international guidelines recommend a maximum whole‐blood donation of 500 mL [4], overfilling is widespread due to the lack of automated weighing systems, frequent power outages and a cultural practice of maximizing volume. In Nigeria, for example, blood collection is often guided by visual estimation, leading to unregulated fill volumes [5].

Overfilling disrupts the critical anticoagulant‐to‐blood ratio, promoting clot formation within the bag [6]. Clots compromise blood quality and—as learned from transfusion of prothrombin complex concentrates—transfusion of activated clotting factors poses significant risks to recipients, including uncontrolled intravascular coagulopathy and thromboembolic complications [7]. Additionally, excessive blood loss during donation increases the risk of orthostatic collapse and seizures in donors, particularly first‐time donors [8, 9]. Although vasovagal reactions after blood donation seem to be less frequent in blood donors of African origin compared to blood donors of European origin [8], blood‐letting of more than 700 mL is far above the internationally recommended 500 mL. These adverse events often deter individuals from becoming regular voluntary donors [9], further worsening the blood supply crisis [10].

Despite its significance, systematic data on overfilling of blood bags and its consequences in sub‐Saharan Africa are scarce. In one of the largest prospective studies of blood donation in the region, the Jini na study [11], we observed that clot formation was the leading cause of blood bag wastage. Of 66,000 blood donations, 1596 were wasted, of which 705 (44.2% of all wasted blood bags) were wasted because of large clots, which did not allow blood transfusion. This is significantly higher than the rates reported in high‐income countries [12]. This finding prompted a deeper investigation into the root causes of clotting, which led to the development of a low‐cost, manual device to prevent overfilling.

We present a novel, low‐cost measuring and mixing (MeMi) device designed to ensure accurate blood collection, prevent overfilling and improve safety for both donors and recipients in resource‐limited settings.

## MATERIALS AND METHODS

### Study design and setting

This was a prospective interventional study conducted across six hospital blood banks in Kano, Nigeria, from January 2025 to January 2026 as follow‐up of the Jini na study. The Jini na study was approved by the Institutional Review Board (IRB) of Aminu Kano Teaching Hospital (AKTH) (approval number: AKTH/IRB/2023/123) and registered under PACTR202310892230317. All procedures were conducted in accordance with the Declaration of Helsinki.

### Data collection and assessment

#### Weight measurement

The weight of 131 consecutive blood bags was measured by research assistants in six hospital blood banks using a calibrated digital scale (accuracy: ±1 g; Kerro Mini Compact Scale Ts‐200 [6 kg/0.1 g]) after collection. An upper weight limit of 612 g and a lower weight limit of 500 g were used. This corresponds to the compound weight of 450 mL whole blood ± 10% (405–495 mL × 1.053 g/L specific gravity for whole blood), citrate phosphate dextrose adenine‐1 anticoagulant and the blood bag weight (80–90 g). This results in an acceptable range of 500–612 g.

#### Clot detection

A systematic assessment of 688 consecutive whole blood bags was performed for macroclots at AKTH. Bags were visually inspected for clots after transfusion, as smaller clots cannot be reliably detected in a full blood bag. The collection centre is located in AKTH. Nurses in the wards collected all blood bags after transfusion. The inspection of the empty blood bags was performed daily by two research assistants who were specifically trained on how to recognize clots to reduce interindividual variability. The bags were then transferred to the transfusion laboratory and re‐assessed by one of the authors (K.M.S.). The whole blood collection bags used were from several different Chinese manufacturers, depending on availability.

#### Intervention

**FIGURE 1:**
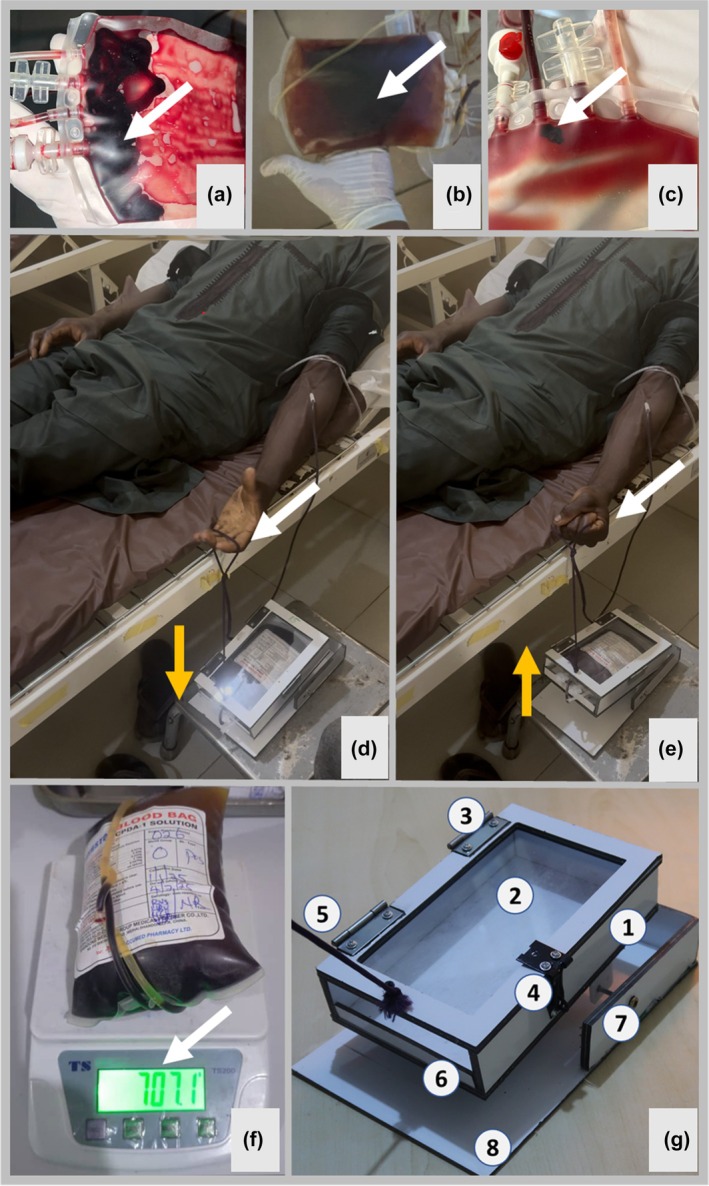
Clots in overfilled blood bags and the measuring and mixing (MeMi) device. Panels (a) and (b) show large clots, indicated by arrows, found in 10% of blood bags after transfusion before introduction of the MeMi device. Panel (c) shows a representative example of small clots found in 1.9% of blood bags after introduction of the MeMi device. Panels (d) and (e) show the MeMi device during blood donation. The blood donor pulls a string at the front of the MeMi device by opening and closing the hand. This moves the box up and down (orange arrows) and secures mixing of the anticoagulant and the donated blood. The video showing the MeMi device in operation is provided as Supporting Information. Panel (f) exemplifies an overfilled blood bag with 707 g (optimal weight range 536–592 g). The mean weight of 131 consecutively assessed blood bags before introduction of the MeMi device was 711 g (range 560–888 g). Panel (g) MeMi device produced locally. (1) Main body of the MeMi device. The dimensions of the MeMi device box determine the volume to which the blood bag can expand. Adding plates reduces the space available at the bottom of the box, thereby decreasing the maximum volume the blood bag can occupy. This allows customizing the device to blood bags from different manufacturers. (2) Glass or plexiglass to monitor filling of the blood bag. (3) Hinges of the lid. (4) Lock. (5) String by which the donor moves the MeMi device box up and down for mixing. (6) Barrier to prevent blood bag slipping out of the MeMi device. (7) Axis on which the MeMi device box is mounted. (8) Base of the MeMi device supports the structure, adds weight and restricts movement. The MeMi device in operation is shown in a movie in Supporting Information.

A manual MeMi device was developed by the authors and implemented. The device consists of a box with a fixed volume (480–500 mL), a string mechanism operated by the donor to ensure anticoagulant‐blood mixing. The donor pulls the string to move the device up and down, promoting mixing, while the box prevents expansion beyond the target volume (Figure 1d,e,g and Video S1). Once the bag fills the MeMi device box, the blood flow stops automatically because the bag can no longer expand. The volume of the box can be reduced by plates added to the bottom of the box to adjust for different blood bag sizes. The MeMi device is produced by a local craftsman using locally available material, aluminium composite panel (ACP; composed of a polyethylene core sandwiched between two aluminium sheets). The filling volume of each device was validated by the first author. For validation, two blood bags were connected: one filled with water and the other empty bag placed in the device. Water was allowed to flow from the filled bag into the empty bag until flow stopped. The final weight was measured to confirm that it fell within 480–500 mL (i.e., weight of the empty bag plus 480–500 g of water). If the volume exceeded this range, plates were added to the base of the device to reduce the filling capacity.

#### Implementation and evaluation

After training of staff, the MeMi device was introduced in the blood donor clinic at AKTH. A total of 723 consecutive blood units, each collected from a different donor, were weighed and inspected for clots following transfusion. Data were analysed using descriptive statistics and 95% confidence intervals (CIs).

#### Statistical analysis

Data were analysed using SPSS (version 28). Descriptive statistics were used to summarize baseline characteristics. CIs were calculated using binomial distribution. Differences in weight of blood bags before and after introduction of the MeMi device were calculated by the Mann–Whitney test. A *p*‐value <0.05 was considered statistically significant.

## RESULTS

### Pre‐intervention data

**FIGURE 2:**
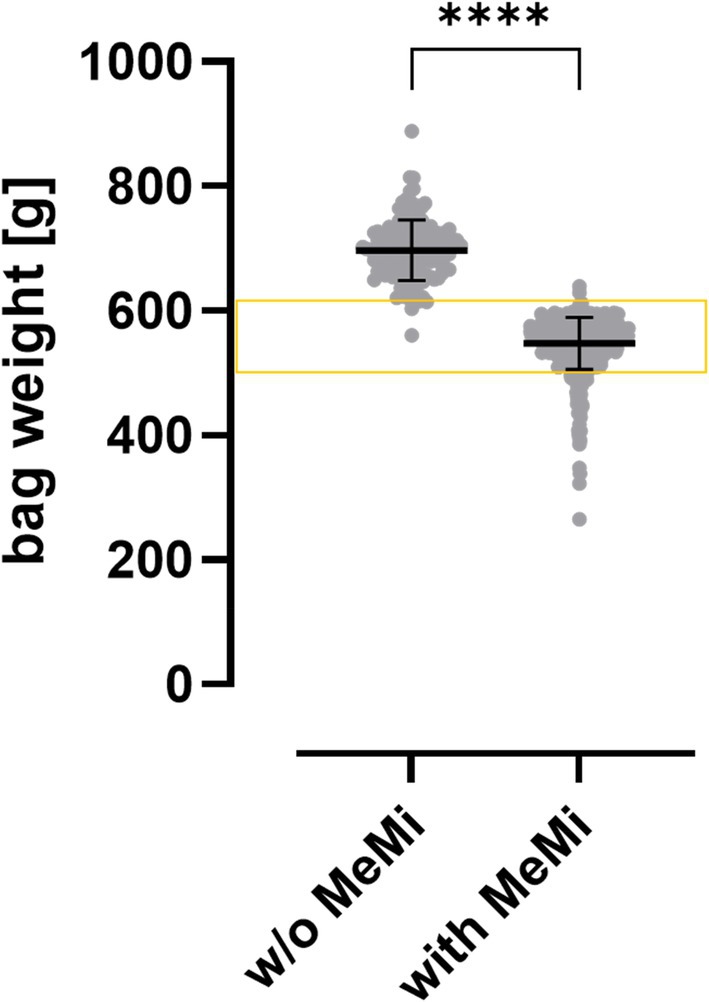
Weight of blood bags before and after introducing the measuring and mixing (MeMi) device. The left column shows the weight of 131 blood bags after blood donations obtained before the MeMi device was introduced; 129 blood bags (98.5%; 95% confidence interval [CI]: 95.2–99.8) exceeded the 612 g limit (weight range 500–612 g). The median weight was 697.6 g (range: 560–888 g). The right column shows the weight of 723 blood bags after blood donations, obtained after the MeMi device was introduced. The weight was considerably less (****p < 0.0001, Mann–Whitney test). Of 723 blood bags, 658 (91%) were within the target weight range. The median weight was 556 g (range 265–639 g). Only 2 blood bags exceeded 612 g, but 63 (8.7%) blood bags were below the lower target weight of 500 g.

Of 131 tested blood bags, 129 (98.5%; 95% CI: 95.2–99.8) exceeded the 612 g upper weight limit. The median weight was 697.6 g (range: 560–888 g) (Figures 1f and 2). When we then systematically assessed 688 consecutive blood bags, macroclots were observed in 72 bags (10.5%; 95% CI: 8.18–12.76) (Figure 1a,b).

### Post‐intervention data

After implementation of the MeMi device, of the 723 blood bags, 658 (91%) were within the target weight range (500–612 g). The median weight was 556 g (265–639 g) (Figure 2). Only 2 blood bags exceeded 612 g, but 63 (8.7%) were below the lower target weight of 500 g. None of the 723 consecutively obtained blood bags was excluded from the analysis. No macroclots were observed. Only 14 of 723 bags (1.9%; 95% CI: 0–5.1) contained small clots post transfusion, which were mainly located close to the tube entrance in the blood bag (Figure 1c).

The MeMi device is easy to use, as it requires no electricity. Although the acceptance of the device by donors and staff was not formally assessed, there were no issues raised in using the device by any donor or staff operating the device.

The device costs approximately €15 to manufacture and was produced by a small local workshop using low‐cost materials.

## DISCUSSION

This study demonstrates that blood bag overfilling is a widespread and a significant problem in resource‐limited settings in sub‐Saharan Africa, including Kano, Nigeria, and likely extends to other regions globally. Overfilling leads to high rates of clot formation, blood wastage and increased risks for donors and recipients. In our study, at baseline, nearly all blood bags exceeded the recommended weight. Furthermore, during the Jini na study, clots were discovered to be the leading cause of blood wastage [11]. This is consistent with other reports from sub‐Saharan Africa [5], but the scale of overfilling and its impact on safety have not been previously quantified in a prospective interventional study.

The development and implementation of the MeMi device provides a simple and low‐cost solution to this problem. The success of the device, namely >90% compliance with weight limits and a 90% reduction in clot formation, demonstrates its effectiveness. Underfilling (~10% of units) most commonly occurred as a result of poor venous access causing interruption of flow. Device handling errors and inadequate donor instruction also contributed; donors who flexed the elbow rather than pulling the MeMi string with their fingers were more likely to sustain venous injury, leading to early termination of collection. Owing to the scarcity of blood donations, all underweight blood bags were used for transfusion [13] as the quality of red cells during storage is not impaired as long as more than 300 mL whole blood was collected as shown using red cell concentrates [13].

Our findings have significant implications for blood transfusion safety in LMICs. Overfilling not only increases the risk of thromboembolic complications in recipients but also contributes to donor morbidity, particularly in first‐time donors [7]. By improving donor safety, the MeMi device can enhance donor retention and strengthen voluntary blood donation programmes, which are critical for sustainable blood supply in sub‐Saharan Africa [1, 2].

Whether clot formation in blood bags causes severe adverse transfusion reactions in the recipient is less clear. No systematic studies exist, as transfusion of clotted blood would be considered unethical. However, transfusion of activated clotting factors has been studied in the context of prothrombin complex concentrates [7] and activated prothrombin complex concentrates with Factor VIII bypassing activity (FEIBA) [7, 14]. Both showed an increase in venous and arterial clots in the case of high doses. Direct transfusion of clots into the recipient is rather unlikely, as transfusion lines in sub‐Saharan Africa are also equipped with a standard 200‐μm filter.

The MeMi device is particularly suitable for low‐resource settings due to its simplicity, affordability and lack of dependence on electricity. It can be implemented in blood drives, walking blood banks, rural maternity clinics and disaster preparedness programmes. Its design allows for local manufacturing and scalability across sub‐Saharan Africa and other LMICs. But it may also be important for disaster preparedness in other regions of the world.

The study has limitations. It was conducted in a single city (Kano), limiting generalizability. Residual clots may be due to the lack of tube strippers, which would ensure complete mixing of anticoagulant in collection tubing. We did not survey the transfusion recipients of blood containing clots for alteration of clotting tests or new thrombotic complications as indicators for coagulopathy induced by a clot in the bag. Future directions are to evaluate the MeMi device in a multi‐centre, randomized trial and to assess its impact on long‐term donor retention and transfusion outcomes. The MeMi device has already been integrated into the transfusion guidelines of the transfusion committee of the Ministry of Health of Kano state, but its integration into national blood safety guidelines and training programmes needs to be further explored and validated.

In conclusion, overfilling of blood bags is a major safety concern in resource‐limited settings, leading to clot formation, increased risk for vasovagal reactions of blood donors, impairment of whole blood quality and blood wastage. The MeMi device is a simple, low‐cost and effective solution that prevents overfilling and improves transfusion safety. It can be locally manufactured and implemented across sub‐Saharan Africa and other low‐resource regions, offering a scalable solution to a critical public health problem.

## CONFLICT OF INTEREST STATEMENT

The authors declare no conflicts of interest.

## Supporting information


**Data S1.** Supporting information.

## Data Availability

Data are available upon reasonable request from the corresponding author. The validated MeMi device is available from Dr. Halima Ismail, National Blood Service Agency, Nigeria, halima.ismail@nbsc.gov.ng.
